# ‘Auspicious liaisons’—evaluating the impact of a liaison geriatrician initiative on older adults psychiatric wards

**DOI:** 10.1093/ageing/afad184

**Published:** 2023-09-22

**Authors:** Peter Swann, Abraham Tolley, Theodoros Paschalis, Daniel Zahedi, Heng C Wong, Viveca Kirthisingha, Eladia Ruiz-Mendoza, Judy Rubinsztein

**Affiliations:** Department of Psychiatry, University of Cambridge, Cambridge, UK; School of Clinical Medicine, University of Cambridge, Cambridge, UK; Cambridge University Hospitals NHS Foundation Trust, Cambridge, UK; School of Clinical Medicine, University of Cambridge, Cambridge, UK; School of Clinical Medicine, University of Cambridge, Cambridge, UK; Cambridgeshire and Peterborough NHS Foundation Trust, Cambridge, UK; North West Anglia NHS Foundation Trust, Peterborough, UK; Cambridgeshire and Peterborough NHS Foundation Trust, OPAC, Cambridge, UK

**Keywords:** dementia, psychiatry, service, older people

## Abstract

**Background:**

Although liaison services in acute hospitals are now the norm, the reverse is not usually available for patients in mental health trusts. Following the introduction of support from geriatricians to older people’s mental health inpatient wards, we wanted to see if this intervention was effective and acceptable to clinicians.

**Methods:**

We performed a retrospective cohort service evaluation on the impact of a liaison geriatrician, using routinely collected data, and assessed acceptability among medical staff by semi-structured interview.

**Intervention:**

Our service introduced regular sessions from consultant community geriatricians across older adults psychiatric wards including a mixture of video conference and face-to-face input.

**Results:**

There was no significant decrease in emergency transfers but there was a significant reduction in length of stay with an associated cost benefit for the service after the introduction of a liaison geriatrician. There was a significant increase in geriatrician consultations and a decrease in specialty consultations to other specialists. There was no change in discharge prescriptions or destination. There was a significant reduction in falls in the intervention arm but not in falls leading to emergency hospital admissions geriatricians gave confidence to psychiatrists of all grades to treat physical health care issues.

**Conclusions:**

A liaison geriatrician service may be a component in reducing length of stay (although there are many others) and improving continuity of care, although it confers no impact on emergency transfers. The intervention was highly acceptable to clinicians.

## Key Points

- There is an unmet need for providing physical healthcare to older adults who are psychiatric inpatients.
- We performed a retrospective cohort evaluation on the impact of a liaison geriatrician on psychiatric wards.
- A liaison geriatrician led to a reduction in length of stay and an improved continuity of care.
- There was no impact on emergency transfers to acute hospital.
- The intervention brings benefits to clinicians including confidence in managing complex cases.

## Introduction

In the year following discharge from an inpatient psychiatric admission, those with schizophrenia or bipolar disorder are twice as likely to die than the general population. Three quarters of these excess deaths are due to medical disorders such as circulatory or respiratory disease [1]. Mortality rates are higher in older people with serious mental illness than those without [2]. The inpatient admission therefore presents a ‘window of opportunity’ to reduce premature deaths [3].

The Royal College of Psychiatrists recommends that older adults with mental illness have access to specialist medical advice whilst on inpatient wards, and compares this with the importance of those in acute hospitals having access to liaison psychiatry [4].

There is evidence that consultant geriatricians reduce length of stay and costs in other specialties such as orthopaedics [5] and the emergency department [6].

More mental health services are integrating sessions with geriatricians or specialist General Practitioners (GPs), but there is limited evidence for the impact liaison geriatricians have on old age psychiatry wards.

Goh *et al.* in 2016 [7] examined medical interventions in an older adults psychiatric ward in Australia following the introduction of a medical resident. They identified high numbers of medical co-morbidities in the inpatient population and found increased medical resident consultations after the introduction of the service but no reductions in emergency transfers.

The study aimed to examine the impact of having a geriatrician on our inpatient psychiatric wards across a range of outcomes. The outcomes measured were: emergency transfers, geriatrician consultations, other speciality consultations, length of stay, changes in non-psychiatric drugs and changes in discharge destination. Falls were measured by looking at falls data from the intervention and comparison period. Qualitatively, we interviewed medical staff to examine their views on this service development.

## Methods

Cambridge and Peterborough NHS Foundation Trust (CPFT) provides physical and mental healthcare to a population of just under a million people in the UK. There are two inpatient mental health services in the trust for older adults, for both functional and dementia patients over the age of 65. The total number of beds in CPFT North is 22 and CPFT South is 32.

The geriatrician in the South of CPFT offered advice for 1 hour every fortnight. Due to COVID-19 pandemic restrictions, this was entirely through videoconferencing but prior to this had been in person. The geriatrician in the North, offered a session (4 hours) to the ward, attending in person and offering support for audits and research. They saw patients individually, accompanied by a trainee, and then joined the ‘dementia’ ward round to discuss their findings with the team. Both geriatricians were happy to be contacted if support was needed between sessions.

We performed a retrospective cohort service evaluation accessed from electronic health records. We compared a period of 6 months prior to and then after the introduction of the liaison geriatrician service (informed by the methodology of Goh *et al.* 2016). Details of how periods for study were chosen are in Supplementary material S1. For each patient, the following outcome metrics were extracted from the relevant section of their record: patient names, admission and discharge dates, unique identifiers, basic demographic information, diagnosis associated with admission, admission and discharge medications, co-morbidities on admission and discharge destinations. Length of stay (LOS) was calculated. Using search terms, emergency transfers, other speciality consultations and geriatrician consultations were identified. To capture the impact of the intervention of polypharmacy, the total number of non-psychiatric drug changes between admission and discharge were recorded as both stopping and starting of medications may have been appropriate. Each researcher extracting data completed a pilot group of cases that were cross checked by other researchers to ensure reliability. The Charlson co-morbidity index (CCI) was calculated retrospectively based on the extracted data, as measures like frailty were not routinely collected on admissions [8] and was used as a surrogate to test whether any differences between groups were due to other factors. The CCI, used previously in psychiatric patients [9], was chosen to capture the impact of co-morbidities. Total number of monthly falls was extracted from incident reports from these periods under review. Patient reported outcomes measures were the question ‘Overall, how was your experience of our service?’ from the NHS family and friends test (Supplementary material S7), performed as a survey at the end of patients stay. Finally, using the personal Social Services Research Unit (PSSRU2019) costs [10], we estimated the cost of the service and inpatient bed days between the comparator and intervention period (Supplementary material S10).

### Semi-structured interview and qualitative analysis

We used purposive sampling to identify participants with knowledge of the intervention, covering clinicians from the range of specialities and seniority impacted. We invited all consultants and trainees (all levels including general practice registrars on psychiatry rotations) practising on the old age wards in June 2020 or the 2 years prior to participate via email invitations. A total of 18 doctors agreed to participate from both services (out of 22, response rate 82%). The survey was primarily conducted by paired interviewers from June 2020 to March 2021. Included were 18 qualified doctors: 3 consultant psychiatrists, 2 consultant geriatricians, 3 senior trainees, 6 core trainees and 4 general practice vocational training scheme. Details of the interview questions are included in Supplementary material S2. Some trainees had been on the ward both prior to and after the geriatricians commenced working on the ward. Answers were recorded in writing by the person not interviewing and interviews were not recorded to enhance free expression.

Data were analysed using a framework approach and two authors (J.R. and P.S.) collaboratively identified themes. The transcripts were reviewed to index phrases to themes, and the number of indexed phrases per theme was recorded. The four most frequently reported themes are reported per question.

### Ethical considerations

Approval was gained from the Research and Development department at CPFT, who agreed this was a ‘service evaluation’ in accordance with the Health Research Authority Guidance and therefore did not require ethics approval.

### Statistical analysis

Demographic, primary and secondary outcomes were compared using unpaired t-tests for normally distributed continuous variables and Mann–Whitney U tests for non-normal distributions. Proportions were compared using x^2^ as per a prespecified analysis plan (using IBM SPSS statistics [11] and JASP [12]). Further post hoc exploratory analysis (incidence rate ratios (IRRs) with R package fmsb [13], Poisson regression in SPSS) was performed to interrogate results for robustness.

## Results

### Retrospective cohort evaluation

There were 222 admissions during the study period (102 prior to intervention and 120 after). Continuous variables (age, length of stay) and frequency counts (number of emergency transfers, geriatrician consultations, other speciality consultations, non-psychiatric drug changes) and CCI were non-normally distributed (Supplementary material S3). There was no difference in age, sex, CCI or diagnostic group between groups in the comparator and intervention periods (Table 1).

**Table 1:** Demographics

Variable	Comparator	Intervention	Significance
Age (median, IQR)	74.5 (10)	76 (12)	0.761^a^
Sex (%)			0.199^b^
– Male	52	43	
– Female	48	57	
CCI (median, IQR)	4.5 (3)	4 (2)	0.498^a^
Diagnosis (%)^c^			0.220^b^
– Dementia	32	36	
– Psychosis	20	15	
– Mood	44	35	
– Other	4	14	
Assessed to lack capacity to consent to treatment during admission (%)			0.52^b^
– Yes	44	48	
– No	56	52	
Admitted under the Mental Health Act			0.241^b^
– Yes	43%	49%	
– No	57%	51%	

^a^Mann–Whitney U-test.

^b^Chi-squared test.

^c^Three from the intervention and one comparator patient had no recorded diagnosis. Other includes anxiety disorders, personality disorders, substance misuse and other non-dementia organic disorders.

Reasons for a geriatrician consultation were diverse but predominantly cardiovascular, infections and electrolyte disturbances. The main reason for emergency transfer was for falls, followed by suspected infections (Supplementary materials S4 and S5).

There was no significant difference in the primary outcome of emergency transfers (U = 5,856, *P* = 0.5, this remained true when admissions were categorised as avoidable, unavoidable or unclear, see Supplementary material S6). There was a significant increase in geriatrician consultations (U = 7,224, *P* = 0.003) and decrease in specialty consultations (U = 4465.5 *P* = <0.001), with small effect sizes. There was no significant difference in patient reported outcomes (Supplementary material S7) or non-psychiatric drug changes (Table 2).

**Table 2:** Difference is primary and secondary outcomes between the comparator and intervention periods

Variable	Comparator	Intervention	Effect size (95% CI)	Significance
Emergency transfers (mean, standard deviation)	0.65 (1.7)	0.44 (0.8)	−0.043 (−0.193, 0.109)	0.5^a^
Length of stay (median, IQR)	78 (78)	52 (55)	−0.238 (−0.376, −0.089)	0.002^a^
Geriatrician consultations (mean, SD)	0.23 (0.543)	0.54 (0.925)	0.18 (0.03, 0.323)	0.003
Speciality consultation (mean, SD)	0.90 (1.05)	0.43 (0.753)	−0.270 (−4.05, −0.124)	<0.001^a^
Patient satisfaction rating (mean, SD)	4.47 (1.06)	4.59 (0.63)	0.031 (−0.130, 0.191)	0.645^a^
Change in non-psychiatric drugs (median, IQR)^b^	2 (3.25)	2 (4)	−0.047 (−0.199, 0.108)	0.549^a^
Change in discharge destination (%)^c^ – Yes – No	29 (25%) 88 (75%)	21 (21%) 81 (79%)	0.79 (0.42–1.49)	0.46^d^

^a^Mann–Whitney U-test. Effect size given by the rank biserial correlation with 95% confidence intervals.

^b^Five intervention and two comparator patients did not have drug changes recorded so based on *n* = 215.

^c^Three cases had no discharge destination recorded so based on *n* = 219.

^d^Chi-squared test. Effect size given as odds ratio with 95% confidence interval.

**Figure 1:**
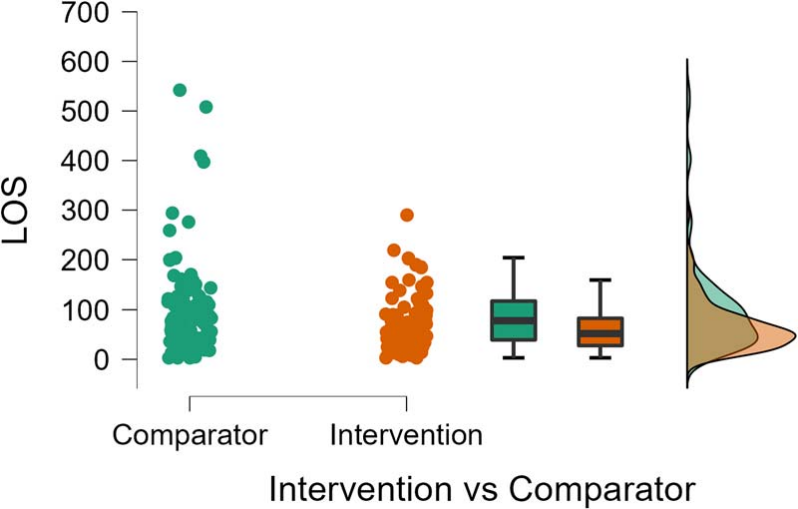
Length of stay dot, box and density plot between the comparator (left) and intervention (right) period. The difference was significant (*P* = 0.002) with a small effect size (U = 4664.5, Mann–Whitney U test).

Length of stay was significantly shorter in the intervention group compared with the comparator group (Figure 1), median 78 vs 52, U = 4664.5, *P* = 0.002) with a small effect size. Observing a number of outliers in the comparator group, this analysis was run again removing all participants with greater than twice the standard deviation of length of stay (7 comparators and 1 from the intervention group) and remained significant (U = 4567.5, *P* = 0.016, effect size −0.192, 95% CI -0.337, −0.038). Exploring the reasons for length of stay with linear regression showed that dementia diagnosis and number of emergency transfers were the main predictors, and geriatricians were more likely to see those with longer lengths of stay (Supplementary material S8). Those managed under the mental health or mental capacity act had prolonged length of stay, but this was due to increased numbers with a dementia diagnosis. Admission under Mental Health Act or management under mental capacity act had no impact on number of emergency transfers (Supplementary material S8).

To ensure effects were not driven by difference in length of stay, we calculated IRRs (Table 3, Rscript Supplementary material S9) with the liaison geriatrician as the exposure and emergency transfers, geriatrician consultations and specialty consultations as the outcome (Table 3). These results showed the same pattern—no significant difference in emergency transfers (estimate = 0.89, 95% CI 0.62–1.23, *P* = 0.522), but increased likelihood of geriatrician consultations (estimate = 3.13, 95% CI 1.94–5.03, *P* < 0.001) and decreased likelihood of speciality consultation (estimate = 0.63, 95% CI 0.45–0.88, *P* < 0.001).

**Table 3:** 

Variable	Incidence rate ratio	95% confidence interval	*P* value
Emergency transfer	0.89	0.62–1.23	0.522
Geriatrician consultation	3.13	1.94–5.03	<0.01
Speciality consultation	0.63	0.45–0.88	<0.01

Incidence rate ratios and 95% confidence intervals for emergency transfers in the comparator period vs the intervention. There is no difference in emergency transfers, a significant increase in geriatrician consultations following the intervention, and reduction of speciality consultations.

The number of falls per month was reduced in the intervention period compared with the comparator period (t-test, *t* = 5.507, *P* = 0.006), but there was no reduction in falls leading to admission (*t* = −1.550, *P* = 0.123). There was also no significant difference in overall patient experience of the ward (U = 4,973, *P* = 0.645, see Supplementary material S7). In our economic analysis, the cost of the geriatrician service was estimated to be £126.45 per admission. The cost for the average difference in length of stay was £14,417.66 per admission (Supplementary material S10).

### Semi-structured interview

The interviews highlight the main challenges of managing physical healthcare on an inpatient psychiatry ward being complexity and co-morbidity, polypharmacy and a lack of senior medical input. Whilst most doctors were fairly confident managing medical issues, psychiatric trainees felt less confident the further away they were from medical training. The primary role of a geriatrician was discussing complex non urgent medical issues, and they added an independent expert medical opinion, both on physical health and shared areas like delirium and dementia. Geriatrician input was perceived to reduce unnecessary referrals and provide reassurance. Reducing acute admissions was commonly mentioned, although this contrasts with the quantitative data above. Psychiatrists and geriatricians each reported increased learning and appreciation from the other speciality. A total of 100% of those surveyed would recommend the liaison geriatrician service, with all respondents either ‘reasonably’ or ‘very’ satisfied on a 5-point Likert scale. Full details of the interview questions and top themes are reported in the supplementary material (Supplementary material S2).

## Discussion

This is the most detailed evaluation of the impact of a liaison geriatrician on older people’s mental health services in the UK. There was no significant reduction in emergency transfers but a significant reduction in length of stay was found. There was significantly increased numbers of geriatrician consultations and a significant reduction in speciality consultations. We did not find any difference in non-psychiatric drug prescriptions or change in discharge destination. There was a reduction in falls, but not in falls leading to emergency transfer. There was no difference in overall patient reported outcomes. Costs for LOS were lower in the intervention group.

Our interpretation is that a liaison geriatrician led to a more holistic continuity of care from a single expert source, rather than the more ad hoc contact of acute services. This may have contributed to a reduction in length of stay, but did not impact emergency transfers, drug prescriptions or discharge destination. The lack of effect may have been due to the primary reasons for transfers being falls and infections, acute events, whilst the geriatrician is focussed on long-term care. Transfers are the culmination of a complex series of events and may be either necessary or unavoidable. Other studies have found increased healthcare contact leads to more admissions [14]. Our study was underpowered to detect a small effect on emergency transfers. Falls were often the presenting complaint leading to admission, but these likely represented multifactorial causes and were not the final discharge diagnosis. LOS was shown to be lower in the intervention group although we are not sure of the causal relationship in our study. There are likely to be numerous unmeasured factors such as impacts and interaction of physical and mental health, social care constraints, differences in how care is given in different teams that may change between the comparator and intervention period.

Input from geriatricians was greatly appreciated by psychiatrists and was mainly around chronic health issues. The trainees liked the continuity of care offered, the pragmatic nature of advice given and the multidisciplinary opinions advised by the same geriatrician. This survey led to the geriatrician in the south offering a weekly consultation rather than fortnightly.

This evaluation’s main strengths were being a real-world sample with pragmatic outcome measures utilised. The excellent response rate to participate in the survey suggested enthusiasm among doctors for the role of geriatricians on the ward.

Limitations identified include patients were not randomised and service evaluations may have limited generalisability to other services. Whilst we postulate that a liaison geriatrician increased geriatrician consultations and reduced other specialty consultations and contributed to reducing length of stay, we cannot claim to have proved causality.

There were significant limitations of a number of outcome measures as we were reliant on information collected at the time. This led to compromises such as the choice of CCI rather than a standardised frailty scale, overall number of medication changes and patient’s overall satisfaction with the service rather than specifically with how their physical health was managed. Future prospective studies are needed to fill these gaps.

We note that interviewers and interpretation were performed by clinicians who worked alongside the service and thus were implicitly supportive of the intervention, which might have influenced answers or interpretation of statements.

The study focussed on the opinions of doctors but did not explore the views of patients, carers nurses and allied health professionals, and this needs to be addressed in future studies.

## Conclusions

This evaluation shows that integrating geriatricians on older people’s mental health wards is an acceptable, cost effective and desirable intervention. Patients received more consultations with a regular geriatrician and fewer with other medical specialities. The intervention period had a reduction in mean length of stay and overall patient falls, however, there will be unmeasured confounders contributing to these results.

The value of geriatrician input is not focussed on preventing acute admissions (such as from falls and infections) but managing co-morbidities and training other doctors.

## Supplementary Material

aa-23-0265-File002_afad184

## Data Availability

The data underlying this article cannot be shared publicly due to the restrictions on routinely collected electronic healthcare records. Deidentified data may be shared upon reasonable request to the authors, however will be dependent on meeting the requirements of CPFT governance.
