# Supplementary material for: ‘Auspicious liaisons’—evaluating the impact of a liaison geriatrician initiative on older adults psychiatric wards
Source: Age Ageing. 2023 Sep 22;52(9):afad184. doi: 10.1093/ageing/afad184 (PMC10789238; doi:10.1093/ageing/afad184)
Supplement: aa-23-0265-File002_afad184 [file aa-23-0265-file002_afad184.docx]

Supplementary materials

Contents:

S1: Methodology for selecting time periods evaluated

S2: Interview questions and key themes

S3: Table - tests of normality

S4: Reasons for geriatrician consultation from interviews and table - ‘Reasons for geriatrician consultation from electronic health records, divided into those listed in RCPsych Report and those not listed’.

S5: Table - Reasons for emergency transfer

S6: Avoidable and unavoidable admissions

S7: Patient reported outcomes

S8: Poisson regression and linear regression of factors predicting length of stay.

S9: Script for calculating incidence rate ratios.

S10: Economic analyses

**Supplement 1: Methodology for selecting time periods evaluated**

Description of the specific times chosen to evaluate the study : lead in time was allowed for the geriatrician to be established for at least a month, and the time of year was maintained as we recognised there may be seasonal variations in presentations which may confound results. Because the geriatrician was introduced at different times at each site, different years were used as baseline comparator and intervention groups for each site. For the North, September 2016 to February 2017 was compared to September 2017 to February 2018. For the South September 2018 to February 2019 was compared to September 2019 to February 2020. All patients admitted between these time periods were included in the study.

**Supplement 2: Interview questions**

What is your name and grade?

Grade when you work on the old age wards and where?

Was this before or after a geriatrician was present? (Rater to note answer next to question above if more than one placement)

What are the main challenges of managing the physical health care of patients on the old age ward?

How confident were you at managing medical issues on the ward?

What do the geriatricians do on the ward?

What do they add to the psychiatric assessment/ formulation?

How many patients do they actually see in person typically?

How many patients do they give advice on during their session typically?

What type of advice typically is asked for from geriatricians?

To psychiatrists only: what have you found most useful about the input of the geriatricians?

To geriatricians only: What do you feel you contribute?

To geriatricians only: What do you feel you learn from being on a psychiatric ward.

What could be improved about this input of geriatricians to psychiatric wards?

Would you recommend the approach of involving a geriatrician on the ward to others?

How satisfied are you with the current service on a likert scale:

Not satisfied at all, limited satisfaction, neutral, reasonably satisfied, very satisfied.

Key themes from interviews:

| Question | Key themes | Supportive quote |
| --- | --- | --- |
| Challenges of managing the physical healthcare of patient on the ward | Complexity and Comorbidity  Polypharmacy  Level of senior support  Psychiatric illness as a barrier to physical healthcare | *‘[Inpatients] are complex, have lots of physical health problems, it was very difficult to know what is attributed to side effects, medication, underlying conditions…’* |
| Confidence in managing medical issues on the psychiatric ward | Reasonably  Quite/fairly confident  Reducing with time away from medicine  Not very confident | *‘Never done GP/ A and E so feel less confident than GP colleagues. A challenge.’* |
| Role of geriatricians on ward | Discussing complex non urgent patients  Doing their own ward round  Seeing patients in person  Email contact outside of sessions | ‘[the geriatrician] was there to go over difficult cases, you had a mini MDT, you bring up patients you have difficulty managing’ |
| What does the geriatrician add to psychiatric assessment or formulation? | Independent view in diagnosing dementia/delirium  Expert medical opinion  Physical health as a precipitating or maintaining factor in mental illness  Limited contribution to psychiatric formulation | *‘Linked physical and mental health problems’* |
| Number seen in person per week? | North mean 5.8, South 0.4 | NA |
| Remote advice per fortnight: | North mean: 7.5 South 5.7 | NA |
| Benefit of geriatrician input (perspective of psychiatrists) | Avoiding unnecessary referrals and acute admissions  Expert physical healthcare  Learning and training  Reassurance | *‘useful to go the same person’*  *‘we developed confidence in dealing with physical health side, this did not just help us on the old age ward but on the on call rota’* |
| Benefits of geriatrician input (perspective of geriatricians) | Support and reassurance  Increased quality of care  Reduced referrals  Continuity of care | *‘…continuity of care, confidence, and good fun!’* |
| Learning from geriatrician’s perspective | Learning more about mental health  Remarkable response to ECT  Increase use of allied health professionals in mental health services  Improve own practice | *‘understand what we can do and what we can’t do’* |
| Improvements suggested | More regular contact  Seeing patients in person (rather than remote)  Increased opportunities for teaching and support for research | *‘We get more skilled and need less help over time. They (the geriatricians) will learn about complex psychiatric patients too, so win win’* |

**Supplement 3: Tests of normality**

| **Table . Tests of Normality** | | | | | | |
| --- | --- | --- | --- | --- | --- | --- |
|  | Kolmogorov-Smirnov^a^ | | | Shapiro-Wilk | | |
|  | Statistic | df | Sig. | Statistic | df | Sig. |
| LOS | .160 | 222 | <.001 | .728 | 222 | <.001 |
| Age | .071 | 222 | .009 | .981 | 222 | .005 |
| CHI | .147 | 222 | <.001 | .940 | 222 | <.001 |
| Emergency transfer | .343 | 222 | <.001 | .395 | 222 | <.001 |
| Geriatrician consultations | .422 | 222 | <.001 | .561 | 222 | <.001 |
| Speciality consultations | .339 | 222 | <.001 | .716 | 222 | <.001 |
| Drug changes | .197 | 215 | <.001 | .861 | 215 | <.001 |
| a. Lilliefors Significance Correction | | | | | | |

**Supplement 4: Reasons for geriatrician consultations from interviews and retrospective cohort evaluation.**

**S4 Table 1:** Reasons for geriatrician consultation from interviews

| **Physical health care topic** | **No of respondents listing** |
| --- | --- |
| Heart failure/cardiovascular | 10 |
| Hypertension | 9 |
| Medications | 8 |
| Requesting and interpreting investigations | 7 |
| Falls | 7 |
| Diabetes Mellitus | 6 |
| Chronic conditions | 6 |
| Appropriate escalation to specialist care/allied health professional | 6 |
| Hypotension | 5 |
| Atrial fibrillation | 3 |
| Differential diagnosis | 3 |
| Dermatology | 3 |
| Weight loss/gain | 2 |
| Parkinson’s disease | 2 |

**S4 Table 2:**  Reasons for geriatrician consultation from electronic health records, divided into those listed in RCPsych Report and those not listed.

From RCPsych report

| **Reason for consultation** | **Count** |
| --- | --- |
| Cardiovascular (including postural hypotension) | 24 |
| Electrolytes | 8 |
| Falls | 5 |
| COPD/respiratory | 3 |
| Parkinson’s (including tremor) | 3 |
| Pain | 2 |
| Polypharmacy | 2 |
| Diabetes | 1 |
| Hyponatraemia | 1 |
| VTE | 1 |
| End of life | 1 |
| Cognitive impairment | 0 |
| Sensory impairment | 0 |
| Oral health | 0 |
| Cerebrovascular | 0 |
| Incontinence | 0 |
| Constipation | 0 |
| Cancer | 0 |
| Hypernatraemia | 0 |
| Pressure areas | 0 |
| Frailty | 0 |

**S4 table 3:** Other reasons not on RCPsych report

| **Reason for consultation** | **Count** |
| --- | --- |
| Infection | 17 |
| Haematology | 4 |
| Leg swelling | 3 |
| Other endocrine | 2 |
| Neuroimaging decision | 2 |
| Weight loss | 2 |
| Other GI | 2 |
| Rheumatology | 2 |
| Mobility | 1 |
| Opthalmology | 1 |
| Gynaecology | 1 |
| Rash | 1 |

**Supplement 5: Reasons for emergency transfers**

**Reasons for emergency transfer (descriptive counts) - note these were presentations not final diagnosis, so falls may represent a myriad of pathologies.**

| **Reason for consultation** | **Count** |
| --- | --- |
| Fall | 44 |
| Infection | 22 |
| Cardiovascular | 14 |
| Urology | 8 |
| Change in mental state | 5 |
| Poor oral intake | 5 |
| Respiratory | 5 |
| Cerebrovascular | 4 |
| Surgical | 4 |
| Electrolyte abnormalities | 4 |
| DKA | 2 |
| Overdose | 2 |
| Endocrine | 2 |
| Constipation | 1 |
| Seizure | 1 |
| Choking | 1 |
| Injury | 1 |
| Headache | 1 |

We reviewed the data on the reasons for geriatrician consultation and emergency admission at an individual level to see if the reasons for consultations overlapped with the reasons for acute transfers. We did not have the temporal relationship between the two events for all cases so are unable to say if the geriatrician review preceded or followed the admission.

Around 28% of admissions were in individuals who had geriatrician consultations for closely related issues, whilst 72% were unrelated. The related issues were primarily falls (21 falls across 8 individuals), with other paired admissions-consultations due to infection, electrolyte disturbance and cardiovascular disease, including management of cardiovascular risk factors post myocardial infarction).

In comparison, there were also numerous falls in individuals not reviewed by the geriatrician for this issue (21 falls across 17 individuals). The remainder of unmatched admissions were for suspected sepsis/infections, cerebrovascular events, and chest pain.

**Supplement 6:** Exploring whether the liaison geriatrician reduced ‘avoidable’ admissions.

We categorised each admission into either ‘avoidable’, ‘unavoidable’ or ‘unclear’ based on the clinical impression of the assessing doctor. For instance, ‘stroke’, ‘sepsis’, ‘chest pain with ECG changes’, and ‘seizures’ were considered unavoidable, most falls and infections without further emergency features ‘unclear’ and ‘dehydration’ potentially avoidable.

There were no reductions in emergency transfers of any kind in the intervention compared to comparator period by ANOVA.

| Admission category | F value | p value |
| --- | --- | --- |
| Avoidable | 0.586 | 0.445 |
| Unavoidable | 0.224 | 0.636 |
| Unclear | 1.323 | 0.251 |

**Supplement 7:** Patient reported outcome measures

Percentage of patients rating their overall experience of the ward as either ‘Very good’, ‘good’, ‘Neither’, ‘poor’, ‘very poor’, or ‘don’t know’ from the friends and family test conducted as a survey prior to discharge. Overall numbers of individuals completing the survey were 110 in the comparator group and 92 in the intervention.

After removing the ‘don’t know’ answers, the answers were converted to a numerical scale (Very good = 5, good = 4, Neither = 3, poor = 2, very poor = 1) and results before and after the intervention compared with Mann-Whitney U test. There was no significant difference in overall patient experience before and after the intervention (p = 0.645), see below.

|  | | | | | | | |
| --- | --- | --- | --- | --- | --- | --- | --- |
|  | | **W** | | **df** | | **p** | |
| Rating |  | 4973.000 |  |  |  | 0.645 |  |
|  | | | | | | | |
| *Note.*  Mann-Whitney U test. | | | | | | | |

Descriptives

*Descriptives Plots*

Rating


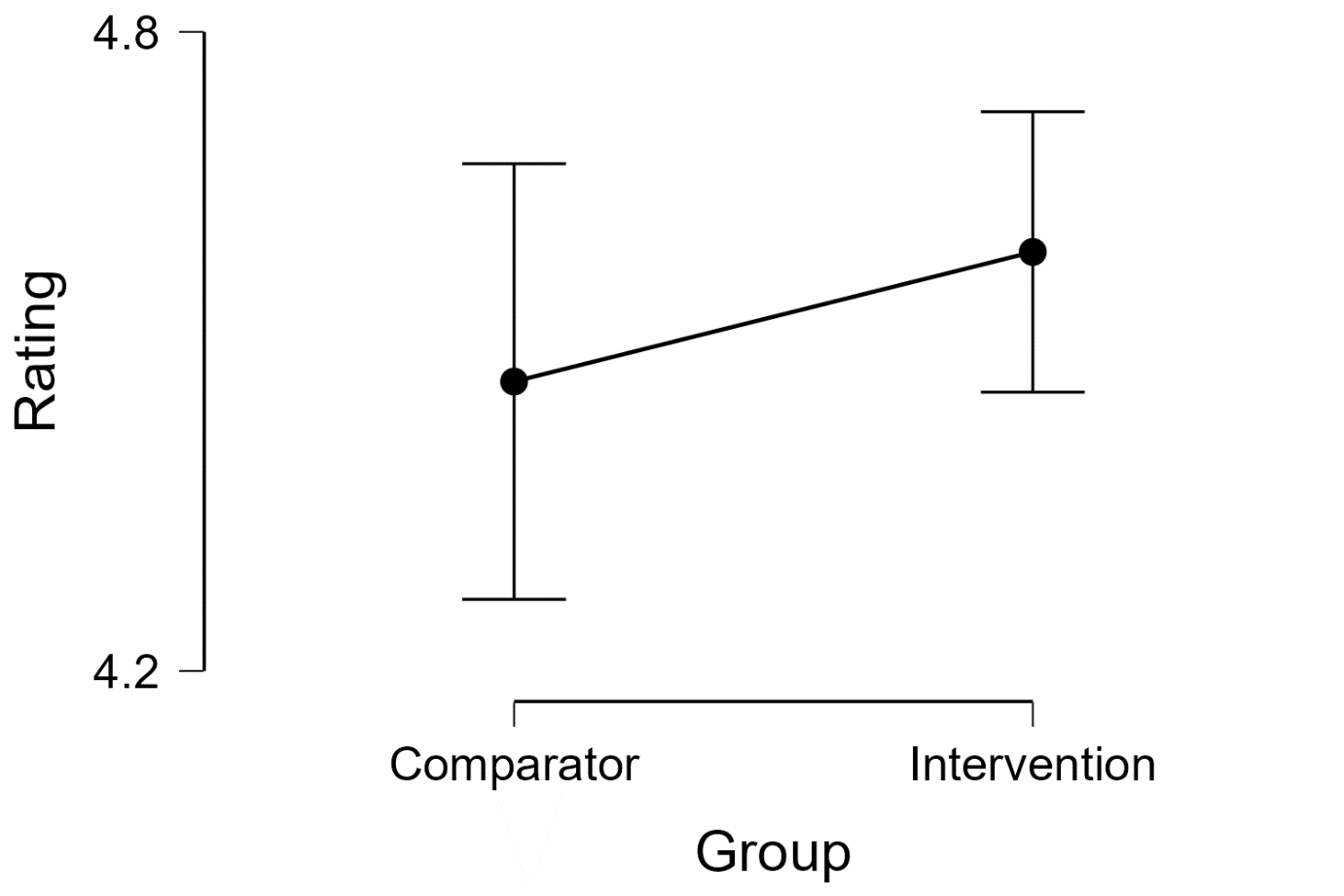


**Supplement 8: Reasons for length of stay**

S2. Poisson regression models were performed in SPSS (IBM Corp. Released 2021. IBM SPSS Statistics for Windows, Version 28.0. Armonk, NY: IBM Corp) using intervention group, age, length of stay and Charlson comorbidity index as predictors and emergency transfers, geriatrician consultations, and speciality consultations as outcomes were performed (table 4). This showed that age, length of stay and CCI predicted emergency transfer (Exp(B) = 1.041, p = 0.04, Exp(B) = 1.005, p<0.001, Exp(B) 1.179, p = 0.006 respectively) but not the liaison geriatrician intervention (Exp(B) = 0.945, p = 0.778). The intervention predicted increased geriatrician consultations and reduced speciality consultations (Exp(B) = 3.126, p<0.001, Exp(B) = 0.508, p<0.001, respectively) and length of stay also predicted geriatrician consultations with a small effect (Exp(B) = 1.005, p <0.001).

**Table (results - Poisson regression)**

| Variable | Comparator vs intervention | Age | Length of stay | Charlson Comorbidity index |
| --- | --- | --- | --- | --- |
|  | Exp(B), p value | Exp(B), p value | Exp(B), p value | Exp(B), p value |
| Emergency transfer | 0.945, 0.778 | 1.041, 0.04* | 1.005, <0.001* | 1.179, 0.006* |
| Geriatrician consultation | 3.126, <0.001* | 0.979, 0.224 | 1.005, <0.001* | 0.975, 0.744 |
| Speciality consultation | 0.508, <0.001* | 0.996, 0.745 | 1.001, 0.158 | 1.051, 0.375 |

**Linear Regression of variables predicting length of stay.**

We conducted a linear regression to explore which factors predicted longer length of stay. This identified diagnosis of dementia or psychosis, number of emergency transfers, and that the geriaitrician was more likely to see those with a longer stay. Inclusion of comparator and intervention status improved the adjusted R^2^ from 0.152 to 0.201 and was significant (p<0.001) suggesting the liaison geriatrician service explained 5% of the variance in length of stay. This also highlights how much of length of stay was driven by unmeasured variables (diagnosis code 1 = dementia, code 2 = mood disorders, code 3 = psychotic disorders).

**Linear Regression**

| **Model Summary - LOS** | | | | | | | | | |
| --- | --- | --- | --- | --- | --- | --- | --- | --- | --- |
| **Model** | | **R** | | **R²** | | **Adjusted R²** | | **RMSE** | |
| H₀ |  | 0.000 |  | 0.000 |  | 0.000 |  | 75.775 |  |
| H₁ |  | 0.490 |  | 0.240 |  | 0.207 |  | 67.487 |  |
|  | | | | | | | | | |

| **ANOVA** | | | | | | | | | | | | | |
| --- | --- | --- | --- | --- | --- | --- | --- | --- | --- | --- | --- | --- | --- |
| **Model** | |  | | **Sum of Squares** | | **df** | | **Mean Square** | | **F** | | **p** | |
| H₁ |  | Regression |  | 296286.038 |  | 9 |  | 32920.671 |  | 7.228 |  | < .001 |  |
|  |  | Residual |  | 938222.624 |  | 206 |  | 4554.479 |  |  |  |  |  |
|  |  | Total |  | 1.235e+6 |  | 215 |  |  |  |  |  |  |  |
|  | | | | | | | | | | | | | |
| *Note.*  The intercept model is omitted, as no meaningful information can be shown. | | | | | | | | | | | | | |

| **Coefficients** | | | | | | | | | | | | | |
| --- | --- | --- | --- | --- | --- | --- | --- | --- | --- | --- | --- | --- | --- |
| **Model** | |  | | **Unstandardized** | | **Standard Error** | | **Standardizedᵃ** | | **t** | | **p** | |
| H₀ |  | (Intercept) |  | 80.829 |  | 5.156 |  |  |  | 15.677 |  | < .001 |  |
| H₁ |  | (Intercept) |  | 20.317 |  | 54.299 |  |  |  | 0.374 |  | 0.709 |  |
|  |  | CHI |  | -1.872 |  | 3.312 |  | -0.042 |  | -0.565 |  | 0.573 |  |
|  |  | Age |  | 0.684 |  | 0.769 |  | 0.064 |  | 0.890 |  | 0.374 |  |
|  |  | Emergency transfer |  | 13.678 |  | 3.627 |  | 0.242 |  | 3.771 |  | < .001 |  |
|  |  | Diagnosis code (1) |  | 26.430 |  | 8.247 |  |  |  | 3.205 |  | 0.002 |  |
|  |  | Diagnosis code (2) |  | -9.990 |  | 7.704 |  |  |  | -1.297 |  | 0.196 |  |
|  |  | Diagnosis code (3) |  | -30.101 |  | 11.879 |  |  |  | -2.534 |  | 0.012 |  |
|  |  | Geriatrician consultations |  | 19.059 |  | 6.371 |  | 0.190 |  | 2.991 |  | 0.003 |  |
|  |  | Change in discharge destination (1) |  | -2.530 |  | 5.645 |  |  |  | -0.448 |  | 0.655 |  |
|  |  | Intervention vs comparator (1) |  | -17.890 |  | 4.816 |  |  |  | -3.715 |  | < .001 |  |
|  | | | | | | | | | | | | | |
| ᵃ Standardized coefficients can only be computed for continuous predictors. | | | | | | | | | | | | | |

We also demonstrated how diagnosis impact length of stay in ANOVA, as shown in the plots below (A – anxiety, D – dementia, M – mood disorder, O – other including drug and alcohol, anxiety, and personality disorder, P – psychotic disorder)

**ANOVA**

| **ANOVA - LOS** | | | | | | | | | | | |
| --- | --- | --- | --- | --- | --- | --- | --- | --- | --- | --- | --- |
| **Cases** | | **Sum of Squares** | | **df** | | **Mean Square** | | **F** | | **p** | |
| Diagnosis code |  | 98905.436 |  | 3 |  | 32968.479 |  | 6.208 |  | < .001 |  |
| Residuals |  | 1.137e+6 |  | 214 |  | 5311.059 |  |  |  |  |  |
|  | | | | | | | | | | | |
| *Note.*  Type III Sum of Squares | | | | | | | | | | | |

Descriptives

*Descriptives plots*


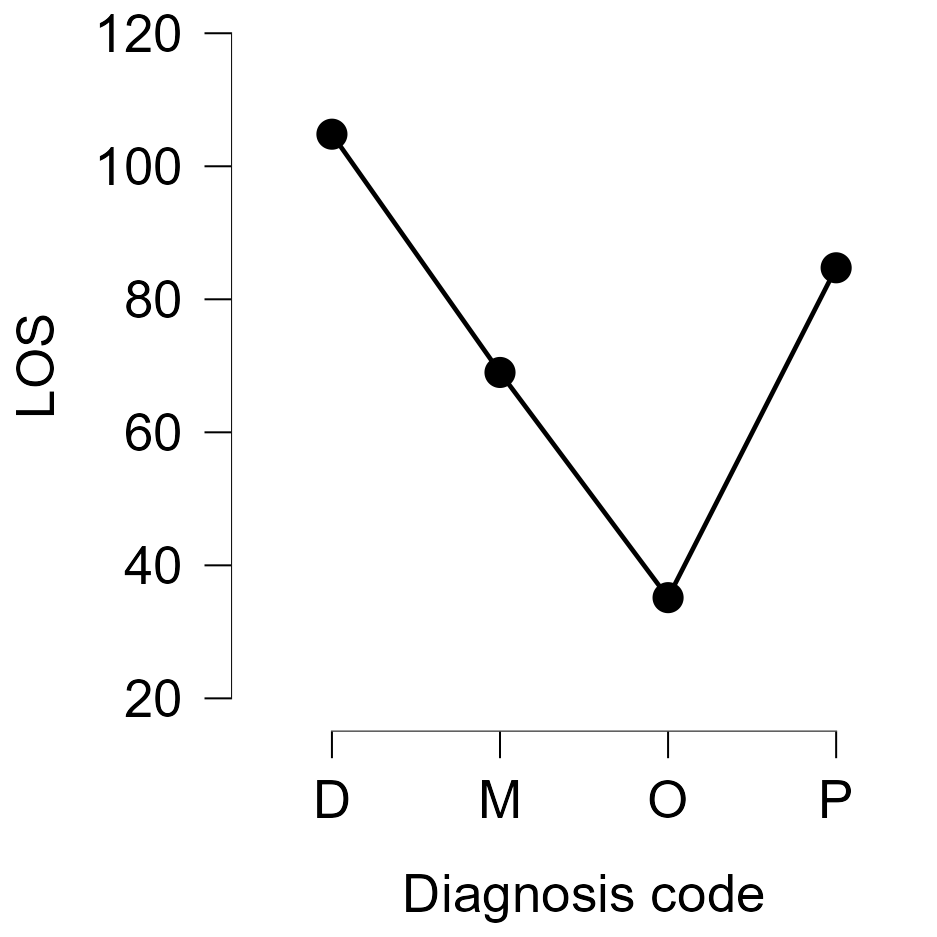


*Raincloud plots*


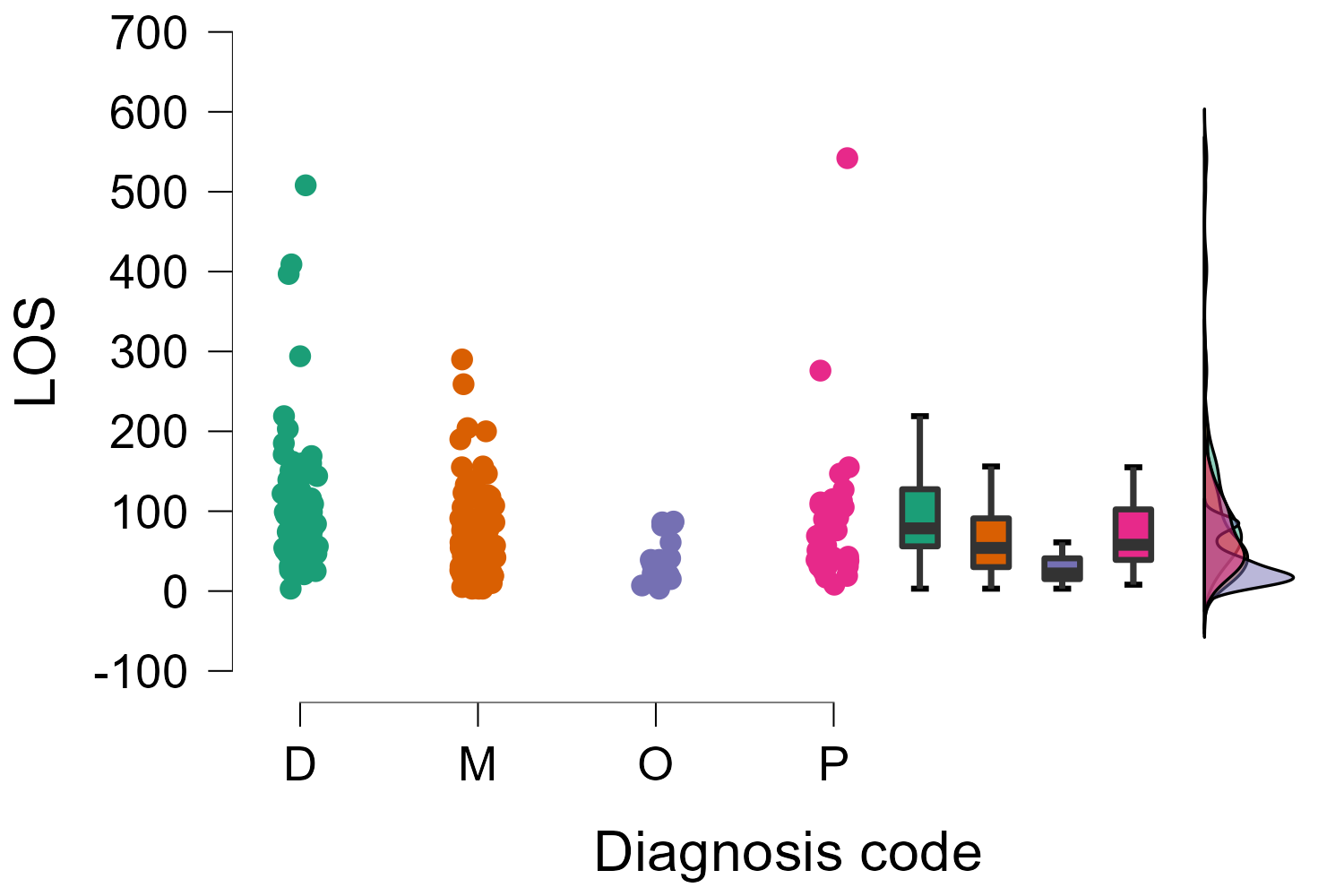


Secondly, we explored whether admission under the mental health act, or individuals assessed to lack capacity to make decisions about influenced length of stay or number of emergency transfers. In separate ANOVAs there was no association between Mental Health Act status and emergency transfers (F = 0.983, p = 0.322). There was a significant association between length of stay and admission under the mental health act (F = 5.597, p = 0.019) but this was no longer significant when controlling for diagnosis (p = 0.524) as more individuals with dementia were both admitted under section and had longer stays. Similarly, being assessed to lack capacity to consent to admission and treatment was not associated with number of emergency transfers (F = 1.220, p = 0.271) and whilst length of stay was associated (F = 9.171, p = 0.003) this was no longer significant when controlling for diagnosis (p = 0.212) for the same reason.

**Supplement S9: RScript for incidence rate ratios**

**S5 Table**

> emergency <- rateratio(53, 66, 921120, 1019286)

Cases Person-time

Exposed 53 921120

Unexposed 66 1019286

Total 119 1940406

> emergency

Incidence rate ratio estimate and its significance probability

data: 53 66 921120 1019286

p-value = 0.5217

95 percent confidence interval:

0.619032 1.275588

sample estimates:

[1] 0.8886112

> geriatrician <- rateratio(65, 23, 921120, 1019286)

Cases Person-time

Exposed 65 921120

Unexposed 23 1019286

Total 88 1940406

> geriatrician

Incidence rate ratio estimate and its significance probability

data: 65 23 921120 1019286

p-value = 7.117e-07

95 percent confidence interval:

1.943791 5.031310

sample estimates:

[1] 3.12727

> speciality <- rateratio(52, 92, 921120, 1019286)

Cases Person-time

Exposed 52 921120

Unexposed 92 1019286

Total 144 1940406

> speciality

Incidence rate ratio estimate and its significance probability

data: 52 92 921120 1019286

p-value = 0.006338

95 percent confidence interval:

0.4451605 0.8787678

sample estimates:

[1] 0.625454

**Supplement S10:** Economic analysis

Acknowledging that in an uncontrolled service evaluation causality cannot be assessed, due to the observation of a reduced length of stay in the intervention group we conducted an economic analysis using the Personal Social Services Research Unit (PSSRU) costs for the year 2019.

We multiplied the length of stay in days for each admission in the study period by the unit cost code for a bed day on the mental health cluster (£424) and summed the total for the comparator and intervention groups. As there were more admissions in the intervention period compared to the comparator (120 vs 102) we corrected the value to be equivalent for 120 admissions.

We then calculated the cost of the geriatrician intervention on a per admission basis. This was based on 5hrs/week consultant physician (at £119/hr) multiplied by the length of stay, divided by 80% of the total bed capacity of the inpatient service, (43 beds). This conservatively assumes an inpatient ward running at on average 80% capacity as not to underestimate the costs of the service).

Estimated costs of length of stay and geriatrician service for the comparator and intervention period

|  | Total cost of bed days for admissions over 6 months | Total cost of geriatrician input | Total cost of length of stay per admission | Total cost of geriatrician input per admission |
| --- | --- | --- | --- | --- |
| Comparator | £4,984,743.53 | NA | £41,539.53 | NA |
| Intervention | £3,254,624.00 | £15,173.49 | £27,121.87 | £126.45 |

This leads to a reduction of £1,730,119.53 for admissions over six months for a cost of £15,173.49, a potential saving of £1,714,946.04. On a per admission basis, it would be a reduction in cost of £14,417.66 for a cost of £126.45, or a potential saving of £14,291.22.

These costs highlight how important length of stay is for costing and are provided as examples based on our local data. This was not a randomised or controlled trial so we cannot attribute the reduction in length of stay to the service. These cost estimates emphasise how important length of stay is in economic evaluation and will hopefully encourage more work assessing the potential economic impact of liaison geriatrician services.
